# Evaluation of Diagnostic Strategies for Identifying SARS-CoV-2 Infection in Clinical Practice: a Systematic Review and Compliance with the Standards for Reporting Diagnostic Accuracy Studies Guideline (STARD)

**DOI:** 10.1128/spectrum.00300-22

**Published:** 2022-06-14

**Authors:** Paula Cremades-Martínez, Lucy A. Parker, Elisa Chilet-Rosell, Blanca Lumbreras

**Affiliations:** a Faculty of Pharmacy, Miguel Hernandez University, Alicante, Spain; b Public Health, History of Medicine and Gynecology Department, Miguel Hernandez University, Alicante, Spain; c CIBER Epidemiology and Public Health (CIBERESP), Madrid, Spain; University of Mississippi Medical Center

**Keywords:** SARS-CoV-2, diagnosis, STARD, COVID-19

## Abstract

We aimed to review strategies for identifying SARS-CoV-2 infection before the availability of molecular test results, and to assess the reporting quality of the studies identified through the application of the STARD guideline. We screened 3,821 articles published until 30 April 2021, of which 23 met the inclusion criteria: including at least two diagnostic variables, being designed for use in clinical practice or in a public health context and providing diagnostic accuracy rates. Data extraction and application of STARD criteria were performed independently by two researchers and discrepancies were discussed with a third author. Most of the studies (16, 69.6%) included symptomatic patients with suspected infection, six studies (26.1%) included patients already diagnosed and one study (4.3%) included individuals with close contact to a COVID-positive patient. The main variables considered in the studies, which included symptomatic patients, were imaging and demographic characteristics, symptoms, and lymphocyte count. The values for area under the receiver operating characteristic curve (AUC)ranged from 53-97.4. Seven studies (30.4%) validated the diagnostic model in an independent sample. The average number of STARD criteria fulfilled was 17.6 (maximum, 27 and minimum, 5). High diagnostic accuracy values are shown when more than one diagnostic variable is considered, mainly imaging and demographic characteristics, symptoms, and lymphocyte count. This could offer the potential to identify individuals with SARS-CoV-2 infection with high accuracy when molecular testing is not available. However, external validation for developed models and evaluations in populations as similar as possible to those in which they will be applied is urgently needed.

**IMPORTANCE** According to this review, the inclusion of more than one diagnostic test in the diagnostic process for COVID-19 infection shows high diagnostic accuracy values. Imaging characteristics, patients’ symptoms, demographic characteristics, and lymphocyte count were the variables most frequently included in the diagnostic models. However, developed models should be externally validated before reaching conclusions on their utility in practice. In addition, it is important to bear in mind that the test should be evaluated in populations as similar as possible to those in which it will be applied in practice.

## INTRODUCTION

There is a wide variety of tests available to detect present or past SARS-CoV-2 infection, both for clinical use and public health applications. Most tests for detecting current infection have focused on reverse transcriptase quantitative PCR (RT-qPCR) assays. However, these tests have limitations, such as the time required to perform them and the presence of false negative results of more than 30% (1), particularly when those tested are in the early stages of infection and/or asymptomatic. Given that asymptomatic patients have been shown to account for up to 80% of those infected (2), and yet can transmit the virus (3), tests need to provide rapid results with sufficient sensitivity and specificity to inform prevention and social isolation measures. The use of rapid diagnostic tests that primarily detect the virus antigen has been widely implemented in practice as an effective strategy to provide a prompt result in a matter of minutes. These tests have similar issues with sensitivity and a limitation in that a positive result must be confirmed by RT-qPCR testing, which makes the implementation of these diagnostic strategies challenging in settings where these molecular tests are not available (4). In addition, in some clinical situations it is necessary to detect the presence of SARS-CoV-2 infection immediately, such as in patients with signs or symptoms of COVID-19, in order to initiate treatment or identify asymptomatic cases and interrupt SARS-CoV-2 transmission when community risk or transmission levels are high. Thus, new effective strategies for diagnosis and detection need to be developed.

A fundamental aspect to consider is that the diagnostic performance of a test is directly related to the context in which it is performed, which needs to be considered when interpreting the results, in addition to the characteristics of the test itself (5). This context will greatly influence the probability or likeliness of the test being positive before the test is undertaken (pre-test probability). For example, a positive test result in individuals who are close contacts of confirmed cases, or in individuals with symptoms suggestive of COVID-19 can give a starkly different predictive value compared to a screening test carried out on an asymptomatic individual undertaking for travel purposes (6).

A previous systematic review assessed the diagnostic accuracy and methodological quality of studies evaluating rapid diagnostic tests for point-of-care antigen detection as well as molecular-based tests (7). In this review, the authors stated that prospective evaluations of rapid tests for COVID-19 infection in clinically relevant settings were urgently needed. In addition, these studies did not include sufficient information on relevant variables such as the patient's symptomatology and the time since symptom onset or exposure, among others. Due to this lack of data, it is not possible to know whether the results obtained can be applied in the different clinical scenarios, i.e., symptomatic, mildly symptomatic, asymptomatic, hospitalized, or for public health use. The authors also concluded that these studies should conform to the Standards for the Reporting of Diagnostic Accuracy Studies (STARD) guideline to enhance the critical appraisal and reproducibility of the studies (8). Similar to other clinical studies, diagnostic accuracy studies are at risk of bias, mainly due to methodological shortcomings in the recruitment of participants, collection of data, execution or interpretation of the test, or the analysis of the data. As a result of these biases, estimates of the sensitivity and specificity of the test compared to the reference standard may not be correct, negatively affecting patient management and the healthcare system. STARDs guideline was developed in 2003 and updated in 2015 to facilitate more complete and transparent reporting of diagnostic accuracy studies to reduce the probability of bias. This guideline contains a checklist of items that should be reported in these studies.

Another more recent review that evaluated studies on the diagnostic accuracy of RT-qPCR-based and other nucleic acid amplification tests showed that these studies had a high risk of bias due to an incorrect study design (according to the aims). This review also highlights the need to use standardized guidelines for study development (9).

These previous reviews focus on the evaluation of individual molecular diagnostic tests applied to the diagnosis or detection of SARS-CoV-2. Given the limitations of molecular testing, a number of clinical tests are usually combined to obtain a final diagnosis. In addition, depending on the clinical setting, different diagnostic processes may exist. For instance, in clinical practice, to achieve adequate diagnostic sensitivity and specificity, a combination of molecular tests, to detect the presence of the virus, and other diagnostic tests such as imaging or biochemical tests, is recommended (10). Although according to a recent systematic review (11), the absence or presence of signs or symptoms were not sufficiently accurate to rule out or suspect COVID-19, combining them with other tests could increase diagnostic accuracy.

Therefore, it is necessary to know the available evidence related to any diagnostic strategy evaluated in practice before getting a molecular diagnosis to better detect SARS-CoV-2 infection. Given the potential for biases shown in previous systematic reviews on molecular tests aimed at detecting COVID-19, it is also necessary to assess their adherence to the STARD guideline.

The aim of our study was to evaluate the available evidence on different diagnostic strategies for identifying SARS-CoV-2 infection before the availability of molecular test results, as well as the reporting quality of the available studies through the application of the STARD guideline.

## RESULTS

### Search results.

**FIG 1:**
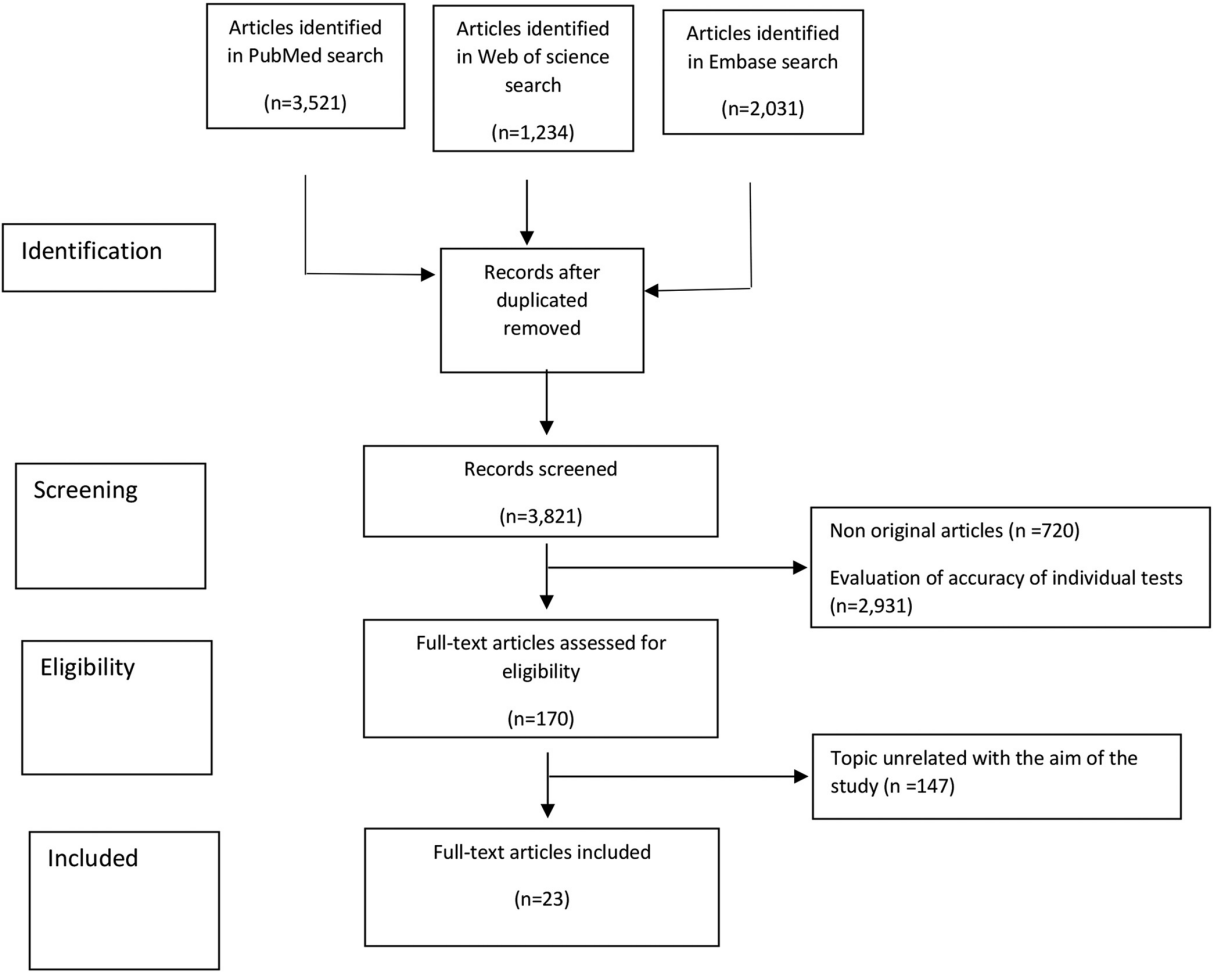
Flow chart of the inclusion of articles in the systematic review, according to Preferred Reporting Items for Systematic Reviews and MetaAnalyses (PRISMA) recommendations.

A total of 3,821 abstracts were reviewed. Of these, 170 studies were included for full text review, and 23 (12–34) met the inclusion criteria (Fig. 1).

### Characteristics of included articles.

Table 1 shows the characteristics of the included studies, which have been classified according to the type of population included: (i) Patients with suspected SARS-CoV-2 infection (16, 69.6%) (12–27), (ii) Patients diagnosed with SARS-CoV-2 (6, 26.1%) (28–33), and (iii) other studies (1, 4.3%) (34).

**TABLE 1:** Description of the main characteristics of the studies included in the systematic review (study characteristics, setting, and population**)**^*a*^

Reference	Yr	Country	Study design	Aim	Setting	Sample size	Gender	Age	Inclusion criteria	Exclusion criteria
Symptomatic patients with suspected COVID 19 infection										
Guo X (12)	2020	China	Retrospective cohort study	To develop an integrated multi-feature predictive model based on random forest to differentiate COVID-19 from seasonal flu and pneumonia caused by other common respiratory viruses.	Two hospitals	105 patients: (a) Discovery cohort, 50 patients and (b) Validation cohort, 55 patients.	Discovery cohort: males, 60%; validation cohort: males, 60%	Age range Discovery cohort: 25-31 yrs (23.1-62.5); Validation cohort: 31-39 yrs (25.0-52.3)	Suspected COVID-19 patients who presented to hospital.	NA
Langer T (13)	2020	Italy	Retrospective cohort study	To develop a machine learning model to predict the results of RT-qPCR for SARS-CoV-2 using only basic clinical, radiological and routine laboratory data at hand in all emergency departments (training and testing and k-fold cross-validation protocol)	Emergency Department at Niguarda Ca′ Granda, Milan, Italy.	199	Males 63.8%	Median : 65 [46–78]	Symptoms of presentation compatible with COVID-19 (fever, sore throat, cough, dyspnoea, chest pain, headache, syncope, asthenia, arthralgia, diarrhoea, nausea and vomit)	Age < 12 yrs and absence of evaluation of the leukocyte formula (defined as percent- ages of the five types of leukocytes: neutrophils, lymphocytes, eosinophils, basophils and monocytes) in the emergency department.
Hermans JJR (14)	2020	The Netherlands	Prospective cohort study	To construct a predictive machine learning model based on chest CT and additional data to improve the diagnostic accuracy of chest CT (10-fold cross validation).	Franciscus Gasthuis & Vlietland hospital in Rotterdam and Schiedam, the Netherlands, which has a level 2 trauma center with 48,000 visits annually at the Emergency Department.	319 patients	Males RT-qPCR+: 55.6%; -RT-qPCR-: 44.6%	Median (IQR) RT-qPCR+: 59 (50-68); RT-qPCR-: 62 (44-75)	Consecutive patients who visited the emergency department between March 27 and April 20, 2020, (a) age ≥ 18 yrs; (b) suspected infection with COVID-19 in combination with at least one of the following: (i) new respiratory symptoms persisting for ≤ 2 wks and present during the last 24 h, (ii) saturation ≤ 94% and/or respiration rate ≥ 20/min and/or abdominal complaints, and (iii) a high clinical suspicion even in the absence of symptoms; and (c) RT-qPCR and chest CT performed within 24 h after each other.	(a) Previously confirmed COVID-19 infection; (b) instability defined as a peripheral oxygen saturation < 92% despite 5 l of oxygen and/or a systolic blood pressure < 90 mmHg; (c) principal presentation due to high energetic trauma, thrombolysis, or acute coronary syndrome; (d) pregnancy; and (e) non-interpretable first RT-qPCR result.
Yang HS (15)	2020	USA	Cross-sectional study	To develop a machine learning model integrating age, gender, race and routine laboratory blood tests, which are readily available with a short turn-around time.	New York Presbyterian Hospital/Weill Cornell Medicine (NYPH/WCM) and New York Presbyterian Hospital/Lower Manhattan Hospital (NYPH/LMH) during March 11 to April 29.	1898 patients: Training set: 1,402 positive and 1,954 negative patients; Validation set: 496 positive and 968 negative patients.	NYPH/WCM: 46.4% and NYPH/LMH: 43.31%	NYPH/WCM: Mean, 56.44 (SD, 19.46) and NYPH/LMH: 56.20 (20.81)	Patients that received a COVID-19 RT-qPCR test in the hospital.	Patients < 18 yrs old, patients who had indeterminate RT-qPCR results, and patients who did not have laboratory results within two days prior to the completion of RT-qPCR testing
AlJame M (16)	2021	Brazil and Italy	Cross–sectional study	To propose a machine learning prediction model to accurately diagnose COVID-19 from clinical and/or routine laboratory data.	Albert Einstein Israelita Hospital located in São Paulo, Brazil and San Raffaele Hospital, Milan, Italy	5923	NA	NA	Patients admitted to hospitals from 28 March to 3 April 2020 in Brazil, from the end of February 2020 to mid-March 2020 in Italy	NA
Tschoellitsch T (17)	2021	Austria	Retrospective cohort study	To evaluate whether machine learning could exclude SARS-CoV-2 RT-qPCR infection using routinely available laboratory values. (internal validation using five-fold cross validation)	Kepler University Hospital in Linz, Austria	1,357	Males: 49.9%	Mean 56.3 yrs (sd 26.6)	Patients with a SARS test performed from 1 March until 30 April	
Du R (18)	2021	China	Retrospective cohort study	To apply machine learning for the task of COVID-19 detection using basic laboratory markers and explore the adjunctive role of chest radiographs	24 hospitals of Hong Kong	Primary cohort: 5230 patients; validation cohort: 605 patients	Males: 56%	NA	Patients with clinical suspicion of COVID-19 infection presenting to the accident and emergency department from the start of the COVID-19 outbreak and had a RT-PCR testing for SARS-CoV-2. The inclusion criteria were: (i) had frontal chest radiographs on the date of the RT-qPCR test, (ii) had laboratory testing done, specifically hematological blood count with or without differential counts, C-reactive protein and lactate dehydrogenase on the date of the RT-qPCR test.	Patients younger than 16 yrs old
Kurstjens S (19)	2020	The Netherland	Retrospective cohort study	To develop an algorithm to rapidly evaluate an individual’s risk of SARS- CoV-2 infection at the emergency department.	Emergency department of three (discovery cohort) and four (validation cohort) different hospitals.	967 patients: (a) discovery cohort: 375 patients, ad (b) validation cohort: 592 patients.	Discovery cohort: COVID-19 negative men 43%; COVID-19 positive men 64.1%. Validation cohort: COVID-19 negative men 53.3%; COVID-19 positive men 63.8%.	Mean (SD). Discovery cohort: COVID-19 negative 62 +-16; COVID-19 positive 70 +-12 Validation cohort: COVID-19 negative 63 +-17; COVID-19 positive 69 +-12	Patients presenting at the emergency department with respiratory symptoms, or suspected COVID-19 infection because of gastro-intestinal complaints (1–2% of this cohort), and subsequent SARS-CoV-2 RT-qPCR	Patients from other departments and patients without any respiratory symptoms or suspicion of COVID-19. For the validation population, patients with missing values or hemolytic samples were excluded.
Tordjman M (20)	2020	France	Retrospective cohort study	To determine a pre-test probability score for SARS-CoV-2 infection.	Four different university hospitals between 10 March and 30 April 2020.	605: Discovery cohort: 200 patients (100 patients and 100 controls). Validation cohort 405 patients.	Discovery cohort: males: patients 65% and controls 45%. Validation cohort: 55% males	Discovery cohort: patients mean 65 (SD, 24) and controls mean 60 (SD, 31). Validation cohort: mean 65	Discovery cohort: outpatients with both RT-qPCR and CT-scan results evaluated for a suspicion of SARS-CoV-2 infection between 10 March and 8 April 2020. Validation cohort: consecutive outpatients suspected of SARS-CoV-2 infection with both RT-qPCR and CT-scan results available.	Discovery cohort: (i) diagnosis still under investigation; (ii) lack of blood test including complete white blood cell count and serum electrolytes; (iii) absence of reported clinical characteristics.
Gupta-Wright A (21)	2021	UK	Retrospective cohort study	To develop and internally validate (bootstrap) a diagnostic risk score to predict risk of COVID-19 (including RT-PCR-negative COVID-19) among medical admissions.	Two hospitals within an acute NHS Trust in London, UK.	4,008 patients	Males: 58.3%	Median, 65 (IQR 57–76)	All patients admitted to medical wards between 2 March and 3 May 2020 with a RT-qPCR test (the decision to test was based on a clinical suspicion).	NA
Vieceli T (22)	2020	Brazil	Cross-sectional study	To develop a useful predictive tool for COVID-19 diagnosis based on clinical, laboratory and image data prior to RT-PCR test confirmation. (Internal validation with bootstrapping)	Hospital de Clínicas de Porto Alegre	100	Males 43%	Median 58 (IQR 40–69.5)	The first consecutive patients aged 18 or older admitted to hospital due to suspected COVID-19 from 17 March to 10 April 2020.	Patients discharged within 24 h of admission
Pardo Lledias J (23)	2020	Spain	Cross-sectional study	To define a score with different clinical probabilities and analyzing the usefulness of repetition of nasopharyngeal smears based on these.	Two COVID internal medicine wards at the University Hospital Marqués de Valdecilla, Santander, Spain (tertiary hospital that covers a population of about 350,000 inhabitants), from March to April 2020.	145	Males 59.9%	Mean, 66.9 (SD,17.8)	Patients admitted for suspected SARS-CoV-2 infection	NA
Trubiano JA (24)	2020	Australia	Cross-sectional study	To report the clinical and epidemiological predictors of COVID-19 from a uniquely derived prospective database and present a point-of-care ready COVID-19 clinical decision tool (internal validation with booststrap).	A COVID-19 rapid assessment screening clinic at Austin Health on 11 March 2020	2935	Males 36.5%	Median, 39 (IQR: 29, 53)	Patients assessed for COVID-19 at a screening clinic due to symptoms (98.3%), contact with known COVID-19 positive patient (17.3%) or overseas travel (24.6%)	NA
Ahmed S (25)	2021	Pakistan	Cross-sectional study	To validate the performance of Corona-Score in a cohort of Pakistani patients pursuing care for suspected infection.	Section of Chemical Pathology in collaboration with the Section of Molecular Pathology, Department of Pathology and Laboratory Medicine, Aga Khan University (AKU), Karachi,	60 patients: 30 RT-qPCR negative and 30 RT-PCR positive.	Males: 41.7%	RT-qPCR positive: mean, 33.1 yrs (SD, 6.5); RT-PqCR negative: mean 60.5 (SD, 16.5)	Suspected COVID-19 cases with respiratory symptoms were recouped from electronical medical records.	NA
Elimian KO (26)	2021	Nigeria States and the Federal Capital Territory.	Retrospective cohort study	To develop and validate the predictive capacity of clinical signs and symptoms about testing positive for COVID-19	Surveillance, Outbreak Response Management and Analysis System (SORMAS) database from 27 February to 27 August 2020.	181,544 patients: derivation set: 90,722; validation set: 90,693.	Males: children, 62.2%; adults, 64%, and elderly, 63.7%	Children (≤17 yrs): 37%; Adults: 61.8%, Elderly: 1.8%.	Patients who were tested for SARS-CoV-2 (according with NCDC COVID-19 standard case definition for suspected cases) with at least one symptom positively recorded. Persons concerned about COVID-19 infection on presentation to testing centers were also included.	No PCR result available; no data on age, sex
McRae AD (27)	2021	Canada	Prospective cohort study	To develop and validate a clinical risk score that can accurately quantify the probability of SARS-CoV- 2 infection in patients presenting to an emergency department without the need for laboratory testing.	32 emergency departments in eight Canadian provinces.(Canadian COVID-19 Emergency Department Rapid Response Network (CCEDRRN) registry)	27,665	Males: 49.5%	Derivation cohort: median 57 yrs (IQR 38–73); validation cohort: median 56 yrs (IQR 37–73)	Consecutive patients aged 18 yrs and older who had a biological sample collected for nucleic acid amplification test (NAAT) on their index emergency department visit or, if admitted, within 24 h of emergency department arrival for known risk factors for SARS-CoV-2 infection, work as a healthcare provider, institutional living, close personal or household contacts with SARS-CoV-2 infection, and symptoms including cough, anosmia or dysgeusia, fever, myalgias and vital signs.	Patients who had a positive SARS-CoV- 2 NAAT within 14 days prior to their emergency department visit, patients with cardiac arrest prior to emergency department arrival and those with missing outcome data.
Confirmed COVID 19 infection										
Gatti M (28)	2020	Italy	Retrospective cohort study	To describe CXR findings and clinical and laboratory characteristics associated with positive and negative CXR.	Emergency department of two Northern Italy hospitals	260	Males 61%	62.8 ± 15.8 yrs	Consecutive patients who were admitted to the emergency department of two Northern Italy hospitals between 1 March and 31 March 2020 with COVID-19 confirmed by RT-qPCR, and who underwent CXR within 24 h of the swab execution.	NA
Góreke V (29)	2021	Israel	Cross-sectional study	To obtain a new feature group based on laboratory findings using the developed algorithm and to detect COVID-19 with a new hybrid classifier based on deep learning that uses the new feature group as input parameters.	Albert Einstein Israelita Hospital	600 patients: 520 diagnosed as COVID-19 and 80 healthy individuals	NA	NA	NA	NA
Arpaci I (30)	2021	China	Cross-sectional study	To predict the COVID-19 positive or negative cases based on 14 clinical features using machine learning classification algorithms. (Internal validation using 10-fold-cross validation)	Taizhou hospital of Zhejiang Province in China	114	Male: 59.6%	Mean, 39.6 (SD 18.8) (range 1–80)	NA	NA
Marateb HR (31)	2021	Iran	Open-cohort study	To design and implement a reliable COVID-19 diagnosis method to provide the risk of infection using demographics, symptoms and signs, blood markers, and family history of diseases to have excellent agreement with the results obtained by the RT-PqCR and CT-scan. (Internal validation using 10-fold-cross validation)	Khorshid Covid Cohort is a hospital-based surveillance study to investigate COVID-19 and non-COVID pneumonia patients since February 2020.	Dataset 1: 634 patients with COVID-19 and 118 with non-COVID-19 pneumonia. Dataset2: 634 patients with COVID-19 and 634 and 598 with non-COVID-19 pneumonia; Dataset 3: healthy individuals from other studies/registries.	Dataset 1: Males: 47.5% (non-COVID-19 pneumonia) and 61.4% (COVID-19) Dataset 2: 45.9%. Dataset 3 not provided.	Dataset 1: Mean,61.7 (SD, 18.3) (non-COVID-19 pneumonia) and 57.0 (15.4) (patients with COVID-19) Dataset 2: Over 60; 89 (14.0%) Dataset 3: not provided	NA	NA
Yousif AY (32)	2022	Iraq	Cross-sectional study	To find a diagnostic method for the COVID-19 virus in Iraq through machine learning algorithms based on blood tests features of Iraqi patients trying to enhance the classification accuracy by selecting the appropriate one for the early prediction of COVID-19.	Samples collected from many private laboratories in Iraq/Baghdad	300 (213 not infected, 87 infected individuals).	NA	NA	NA	NA
Plante TB (33)	2020	USA and Israel	Case-control study	To describe the development of a machine learning model for ruling out COVID-19 using only routinely collected laboratory tests. Furthermore, to assess the AUROC curve of the model with both COVID-19 PCR test results (cases) and prepandemic patients (controls).	Three data sets of patients in emergency department : (a) The Premier Healthcare Database (155 hospitals); (b) Cedars-Sinai Medical Center an 886-bed academic medical center in Los Angeles, CA; and (3) the Beth Israel Deaconess Medical Center a 673-bed academic medical center in Boston.	192,778 patients: training set (12,183) and validation set (172,754); sensitivity analysis (7,842)	Median age decile 50 (IQR 30-60)	Males: 40.5%	Adults aged ≥20 yrs in an emergency department at an included center during one of the prepandemic or pandemic time frames.	Missing a laboratory result included in the model on the day of presentation to the ED or if any of their laboratory results were reported with inappropriate units or incorrect specimen type.
Other										
Banerjee A (34)	2020	Brasil	Cross-sectional study	To use machine learning, an artificial neural network, random forest, and a simple statistical test to identify SARS-CoV-2-positive patients from full blood counts without knowledge of symptoms or history of the individuals.	Hospital Israelita Albert Einstein, at São Paulo, Brazil.	598	NA	NA	Patients who had samples collected to perform the SARS-CoV-2 RT-qPCR and additional laboratory tests during a visit to the hospital.	Patients in semi-intensive unit and ICU

*^a^*AUC, area under the receiver operating characteristic curve; CT, computerized tomography; CXR, chest X-rays; ED, emergency department; ICU, intensive care unit; IQR, interquartile range; NA, not available; SD, Standard deviation.

### (i) Studies including symptomatic patients with suspected SARS-CoV-2 infection.

Of the 16 studies which focused on symptomatic patients with suspected SARS-CoV-2 infection (12–27), seven included techniques related with artificial intelligence (AI) (12–18): five of them used a cohort study, either retrospective (12, 13, 17, 18) or prospective (14), and two used a cross-sectional study (15, 16). The other nine studies developed an algorithm (19, 24, 26) or a probability score (20–23, 25, 27) to diagnose infection by SARS-CoV-2. Four of them used a cross-sectional study (22–25), four used a retrospective cohort study (19–21, 26), and one used a prospective cohort study (27). Most of the studies included symptomatic patients who were admitted to a hospital due to suspected COVID-19; Trubiano JA et al.’s study (24) included patients assessed for COVID-19 at a screening clinic due to symptoms (98.3%), contact with known COVID-19-positive patient (17.3%), or those who had traveled abroad (24.6%), and Elimian KO et al. (26) included symptomatic patients and also those concerned about COVID-19 infection.

The sample sizes included in these studies ranged from 100 to 181,544 patients (90,772 in the derivation set and 90,772 in the validation set).

Tables 2 and 3 show that the main variables included in the models were lymphocyte count (10/16 studies), imaging characteristics (CXR/CT) (9/16 studies), demographic characteristics (8/16 studies), and symptoms (7/16 studies). Other variables were less likely to be included in the models: laboratory routine tests (4/16) and different biochemical measurements (LDH, 6/16 studies; CRP, 6/16 studies; and ferritin, 4/16 studies). In addition, one of the studies (12) included in the diagnostic model the first RT-qPCR result. The models showed different levels for area under the receiver operating characteristic curve (AUC) (ranged 53–97.4).

**TABLE 2:** Description of the variables included in the study, the results obtained and the main conclusions of the study^*a*^

Reference	Variables/tests included	Reference standard	Results	Authors' conclusion
Symptomatic patients with suspected COVID 19 infection				
Guo X (12)	CT scans; pharyngeal swab samples for first RT-qPCR analysis; blood samples (WBC, LYC, and LYP); age, sex, epidemiological record and clinical symptoms (fever, sore throat, cough, fatigue).	First RT-qPCR	An integrated multi-feature model (RT-qPCR, CT features, and LYP) established with random forest algorithm showed the diagnostic accuracy of 92.0% (95% CI: 73.9 - 99.1) in the training set, and 96. 6% (95% CI: 79.6 - 99.9) in the internal validation cohort. The model also performed well in the external validation cohort with an AUC of 93 (95% CI: 79 - 100).	The developed multivariate model based on machine learning techniques could be an efficient tool for COVID-19 screening in nonendemic regions with a high rate of influenza and CAP in the post-COVID-19 era.
Langer T (13)	Age, gender, presence and type of comorbidities, reported symptoms. Vital signs upon admission to the ED (first measurement), presence and type of ventilatory support, routinely performed blood tests, major electrocardiographic characteristics (presence of sinus rhythm and ST abnormalities) and CXR (presence of any type of parenchymal involvement, presence of pleural effusion)	RT-qPCR	The best Machine Learning System reached an accuracy of 91.4% with 94.1% sensitivity and 88.7% specificity.	Basic clinical data might be sufficient for properly trained algorithms to predict with good accuracy the positivity and negativity to SARS-CoV-2.
Hermans JJR (14)	CXR (classified according to the CO-RADS classification), ferritin, leucocyte count, CK, presence of diarrhea and no. of days since onset of disease.	RT-qPCR	The prediction model with CO-RADS, ferritin, leucocyte count, CK, days of complaints, and diarrhea as predictors had a sensitivity, specificity, PPV, and NPV of 89.3%, 93.4%, 90.8%, and 92.3%, respectively. AUC = 93.4%	Combining a predictive machine learning model could further improve the accuracy of diagnostic chest CT for COVID-19. Further candidate predictors should be analyzed to improve our model. However, RT-PCR should remain the primary standard of testing as up to 9% of RT-PCR positive patients are not diagnosed by chest CT or our machine learning model.
Yang HS (15)	Patient demographic features (age, sex, race) with 27 routine laboratory tests	RT-qPCR	The model achieved an area under the AUC of 85.4 (95% CI: 82.9-87.8).	This model employing routine laboratory test results offers opportunities for early and rapid identification of high-risk SARS-CoV-2 infected patients before their RT- PCR results are available.
AlJame M (16)	Clinical tests: AST, ALT, WBC, platelets, CRP, ALP, LDH, monocytes, gender, age.	RT-qPCR	Experimental results show that the proposed DF model has an accuracy of 99.5%, sensitivity of 95.28%, and specificity of 99.96%.	These performance metrics are comparable to other well-established machine learning techniques, and hence, DF model can serve as a fast-screening tool for COVID-19 patients at places where testing is scarce.
Tschoellitsch T (17)	Standard laboratory values: blood count, electrolytes, C-reactive protein, creatinine, blood urea nitrogen, liver enzymes, bilirubin, cholinesterase, and prothrombin time.	RT-qPCR	The machine learning model could predict SARS-CoV-2 test results with an accuracy of 86% and an area under the ROC of 0.74.	Machine learning methods can reliably predict a negative SARS-CoV-2 RT-PCR test result using standard blood tests
Du R (18)	Sex, age, laboratory data: hemoglobin, hematocrit, white blood cells, lymphocytes, monocyte, neutrophil, platelet, CRP, LDH, and chest radiographs.	RT-qPCR	For predicting SARS-CoV-2 infection, the ML model achieved high AUCs and specificity but low sensitivity in all three validation sets (AUC: 89.9–95.8%; sensitivity: 55.5–77.8%; specificity: 91.5–98.3%). When used in adjunction with radiologist interpretations of chest radiographs, the sensitivity was over 90% while keeping moderate specificity.	The study showed that machine learning model based on readily available laboratory markers could achieve high accuracy in predicting SARS-CoV-2 infection.
Kurstjens S (19)	Laboratory measurements (CRP, ALC, ANC, LDH and ferritin), age, sex and CXR/CT	RT-qPCR	The corona-score model yielded an AUC of 91% in the validation population.	The corona-score provides the means for medical professionals to rapidly evaluate SARS-CoV-2 infection status of patients presenting at the ED with respiratory symptoms.
Tordjman M (20)	Demographic characteristics, comorbidities (hypertension, respiratory diseases [asthma, COPD], immunodeficiency, renal insufficiency), clinical symptoms (cough, fever, headache, diarrhea, anosmia, ageusia, oxygen desaturation), and biological tests (WBC, serum electrolytes and RP)	RT-qPCR and/or CT-scan showing signs of COVID-19 pneumonia	In the multivariate analysis, lymphocyte (<1.3 G/L), eosinophil (<0.06 G/L), basophil (<0.04 G/L) and neutrophil counts (<5 G/L) were associated with high probability of SARS-CoV-2 infection, but no clinical variable was statistically significant. The score had a good performance in the validation cohort (AUC = 91.8 (CI: [89.1–94.6]; STD = 0.014) with a Positive Predictive Value of high-probability score of 93% (95%CI: [89–96]). Low-probability score excluded SARS-CoV-2 infection with a Negative Predictive Value of 98% (95%CI: [93–99]).	The PARIS score has a good performance to categorize the pre-test probability of SARS- CoV-2 infection based on complete white blood cell count. It could help clinicians adapt testing and for rapid triage of patients before test results.
Gupta-Wright A (21)	Demographic characteristics (age, sex, ethnicity), CXR, clinical symptoms associated with COVID-19 (cough, fever or shortness of breath), vital signs (NEWS 2 and laboratory bloods (CRP and arterial/venous blood gas)	Patients with a positive SARS-CoV-2 RT-qPCR within 7 days before or after the date of admission and had a discharge diagnosis of COVID-19.	The following variables: age, sex, ethnicity, cough, fever or shortness of breath, NEW 2, CRP, and CXR appearance had moderate discrimination (AUC 83%, 95% CI 82 – 85%), good calibration and was internally validated.	Diagnostic risk scores could potentially help triage patients requiring admission but need external validation.
Vieceli T (22)	Clinical characteristics, PSI/PORT score, comorbidities, laboratory findings and radiographic findings.	RT-qPCR	Variables associated with COVID-19 diagnosis in multivariate analysis were leukocyte count ≤7.7 × 103 mm–3, LDH >273U/L, and CXR abnormality. After bootstrapping, the corrected AUC for this model was 82.7 (95% CI 75–90).	This predictive score that can be easily applied in clinical practice, but it is yet to be validated in larger populations.
Pardo Lledias J (23)	Epidemiological contact, clinical presentation as pneumonia, absence of pneumonia in the last yr, onset of symptoms > 7 days, two or more of the following symptoms -dyspnea, cough or fever- and serum lactate dehydrogenase levels >350 U/L (p < 0.05).	RT-qPCR	A score based on the independent variables yielded an AUC-ROC of 89 (CI95%, 83.1–94.6; p < 0.001). The accuracy of the first nasopharyngeal swabs was 54.9%. Repeating nasopharyngeal swabs two or three times allows to detect an additional 16% of positive cases. The overall accuracy of successive RT-PCR tests in patients with low pre-test probability was <5%.	The pre-test probability score based on epidemiological and clinical data showed a high accuracy for diagnosis of SARS-CoV-2. Repeating nasopharyngeal swabs avoids sampling errors, but only in medium of high probability pre-test clinical scenarios. This score should be externally validated.
Trubiano JA (24)	Clinical data from the data collection tool (baseline demographics, clinical symptoms, clinical observations)	RT-qPCR	The 7 features associated with a positive COVID- 19 test on multivariable analysis were: COVID-19 patient exposure or international travel, myalgia/malaise, anosmia or ageusia, temperature, coryza/sore throat, hypoxia–oxygen saturation < 97%, 65 yrs or older. Internal validation showed an AUC of 83.6.	The clinical decision rule, COVID-MATCH65 has a high sensitivity and negative predictive value for SARS-CoV-2 and can be employed in the pandemic, adjusted for disease prevalence, to aid COVID-19 risk-assessment and vital testing resource allocation. Authors encourage readers to urgently employ and validate COVID-MATCH65 in their own datasets.
Ahmed S (25)	Biochemical data (serum LDH, CRP and ferritin), hematological data (absolute neutrophil and lymphocyte count) and imaging data (presence of infiltrates on chest X-ray).	RT-qPCR	The AUC of Corona-Score in our population of patients was 0.59 (95% CI: 0.45–0.74). Using the cut-off values of 4 originally identified by Kurstjens et al. for their study population, the model displayed 43.3% sensitivity and 70% specificity with an overall accuracy of 56.67%.	Corona Score is an easy-to-use algorithm for identification of COVID-19 patients with respiratory symptoms and needs to be further validated on a bigger sample size. Large multi-center studies across the country are in dire need of time to evaluate the score in overly.
Elimian KO (26)	Clinical signs and symptoms: chills/sweat; cough; breathing difficulty; rapid breathing; runny nose; abdominal pain/diarrhea; gastrointestinal symptoms; chest pain; fatigue; headache; musculoskeletal pain; sore throat; loss of taste; loss of smell; fever.	RT-qPCR	Best individual symptom predictor of COVID-19 positivity was loss of smell in children (AUROC 0.56, 95%; CI 0.55 to 0.56), either fever or cough in adults (AUROC 0.57, 95%; CI 0.56 to 0.58) and difficulty in breathing in the elderly (AUROC 0.53, 95%; CI 0.48 to 0.58) patients.	The predictive capacity of various symptom scores for COVID-19 positivity was poor overall. However, the findings could serve as an advocacy tool for more investments in resources for capacity strengthening of molecular testing for COVID-19 in Nigeria.
McRae AD (27)	Demographics: age, sex, arrival from (home + other, single room + no fixed address + shelter, institutional living, inter-hospital transfer), infection risk, emergency department variables, COVID symptoms, 7-day avg incident COVID-19 cases	NAAT	The score had a c-statistic of 0.838 with excellent calibration. Score cut-offs were identified that can rule-in or rule-out SARS-CoV-2 infection without the need for nucleic acid testing with 97.4% sensitivity (95% CI 96.4 to 98.3) and 95.9% specificity (95% CI 95.5 to 96.0).	The score can identify patients at sufficiently high risk of SARS-CoV-2 infection to warrant isolation and empirical therapy prior to test confirmation while also identifying patients at sufficiently low risk of infection that they may not need testing
Confirmed COVID 19 infection				
Gatti M (28)	Comorbidities (presence of cardiac disease, diabetes, obesity, hypertension, smoke history, ACEi/Sartan or FANS therapy), Clinical data (fever, cough, rhinitis, dyspnea, pharyngodynia, myalgias, asthenia, conjunctivitis, headache, nausea, vomit and diarrhea), laboratory data (WBC, CRP, LDH, hepatic enzymes, CK, blood’s pH, PaCO2) and CXR.	RT-qPCR	The ROC curve procedure determined that CXR+ was associated with LDH > 500 UI/L (AUC = 87.8%), CRP > 30 mg/L (AUC = 83.0%) and interval between the onset of symptoms and the execution of CXR > 4 days (AUC = 75.0%). The presence of two out of three of the above-mentioned predictors resulted in CXR+ in 92.5% of cases, whereas their absence in 7.4%.	CXR has a low sensitivity. LDH, CRP and interval between the onset of symptoms and the execution of CXR are major predictors for a positive CXR.
Góreke V (29)	Laboratory tests routinely collected, age, race, sex, and disease severity subgroups.	RT-qPCR	A development and external validation study of a machine learning model for COVID-19 status using laboratory tests routinely collected in adult ED patients found high discrimination across age, race, sex, and disease severity subgroups. The AUROC for the training and external validation data set was 91% (95% CI 90%–92%).	A machine learning model developed with multicenter clinical data integrating commonly collected ED laboratory data demonstrated high rule-out accuracy for COVID-19 status and might inform selective use of PCR-based testing.
Arpaci I (30)	Laboratory data: hematocrit, hemoglobin, platelets, red blood cells, lymphocytes, leukocytes, basophils, eosinophils, monocytes, serum glucose, neutrophils, urea, CRP, creatinine, potassium, sodium, ASL, ASP.	RT-qPCR	Classification performance indicators were obtained as accuracy of 94.95%, F1-score of 94.98%, precision of 94.98%, recall of 94.98% and AUC of 100%.	Proposed method shows superior performance and can provide more convenience and precision to experts for diagnosis of COVID-19 disease.
Marateb HR (31)	Laboratory data: white blood cell count, neutrophil, lymphocyte, monocytes, eosinophil, basophils, neutrophil-lymphocyte, lymphocyte/monocyte, hemoglobin, hematocrit, mean red blood cell volume, platelet, thrombocytocrit and procalcitonin.	RT-qPCR	The CR meta-classifier is the most accurate classifier for predicting the positive and negative COVID-19 cases with an accuracy of 84.21%.	The results could help in the early diagnosis of COVID-19, specifically when the RT-PCR kits are not sufficient for testing the infection and assist countries, specifically the developing ones that suffer from the shortage of RT-PCR tests and specialized laboratories.
Yousif AY (32)	Demographics: age, gender, occupation; laboratory data: white blood cells, CRP, LDH, PLT, lymphocytes, hemoglobin sodio; symptoms and signs: shortness of breath, PCO2, cough details, decreased appetite, headache, body temperature; other: contact with conformed COVID-19 patients, other comorbidities, sore throat, myalgia, chronic respiratory disease, symptom duration, COPD, weight loss, chills, diarrhea, SatO2	RT-qPCR and chest CT	Sensitivity of 96% (CI, 95%: 94–98), specificity of 95% (90–99), positive predictive value (PPV) of 99% (98–100)], negative predictive value (NPV) of 82% (76–89), diagnostic odds ratio (DOR) of 496 (198–1,245), area under the ROC 0.96 (0.94–0.97), Matthews Correlation Coefficient of 0.87 (0.85–0.88), accuracy of 96% (94–98), and Cohen’s Kappa of 0.86 (0.81–0.91). The AUC on the datasets 2 and 3 was 0.97 (0.96–0.98) and 0.92 (0.91–0.94), respectively. The most important feature was white blood cell count, shortness of breath, and C-reactive protein for datasets 1, 2, and 3, respectively.	The proposed algorithms, thus, a promising COVID-19 diagnosis method, which could be an amendment to simple blood tests and screening of symptoms. However, the RT-PCR and chest CT-scan, performed as the gold standard, are not 100% accurate.
Plante TB (33)	Laboratory data: oxygen content, ferritin, CRP, WBC, LYM, GRA, RBC.	NA	The results show that the best classification accuracy obtained was 0.87, associated with an F1-Score of 0.91.	Machine learning algorithms can be used in conjunction with blood tests in countries with insufficient resources to combat this pandemic.
Other				
Banerjee A (34)	Standard full blood count: hematocrit, hemoglobin, platelets, MPV, RBC, lymphocytes, MCHC, leukocytes, basophils, neutrophils, MCH, eosinophils, MCV, monocytes and RBCDW.	RT-qPCR	Full blood counts random forest, shallow learning and a flexible model predict SARS- CoV-2 patients with high accuracy between populations on regular wards (AUC = 94–95%) and those not admitted to hospital or in the community (AUC = 80–86%). A simple linear combination of 4 blood counts can be used to have an AUC of 85% for patients within the community.	This new methodology has potential to greatly improve initial screening for patients where based diagnostic tools are limited. Further validation will be required to determine if the model can distinguish fully from other pathogens.

a ACEi/Sartan or FANS therapy; ALC, absolute leukocyte count; ANC, absolute neutrophil count; AUC, area under the receiver operating characteristic curve; CK, creatin kinase; COPD, chronic obstructive pulmonary disease; CRP, C-reactive protein; CT, computerized tomography; CXR, chest X-rays; ED, emergency department; LDH, lactate dehydrogenase; LYC, lymphocyte count; LYP, blood lymphocyte percentage; MCHC, mean corpuscular hemoglobin concentration; MCV, mean corpuscular volume; MPV, mean platelet volume; NAAT, nucleic acid amplification test; NEWS 2, National Early Warning Score 2; PaCO2, arterial partial pressure of carbon dioxide; PSI/PORT score, Pneumonia Severity Index for community-acquired pneumonia; RBC, red blood cells; RBCDW, red blood cell distribution width; WBC, white blood cell count.

**TABLE 3:** Variables finally included in the model to diagnose infection with s**ymptomatic patients with suspected COVID 19 infection**^*a*^

References	AUC (IC 95%)	RT-qPCR	Demographic (age, sex)	Clinical characteristics					Image		Whole blood tests				Biochemical measurements							
				Comorbidities	Symptoms	Exposure or international travel	ECG	Days since onset	CT	CXR	RBC	LYP	LYC	WBC	Laboratory routine tests	CRP	LDH	Ferritin	CK	Arterial/venous blood gas	Cholinesterase	Prothrombin time
Guo X (12)	93 (79–100)	X							X			X										
Langer T (13)	90		X	X	X		X			X	X		X									
Hermans JJR (14)	91.4 (87.9–94.9)				X			X		X			X					X	X			
Yang HS (15)	85.4 (82.9–87.8)		X								X	X	X	X	X							
Aljame M (16)	Accuracy 99.5%		X								X	X	X	X	X							
Tschoellitsch T (17)	0.74										X				X	X					X	X
Du R (18)	89.9–95.8		X							X	X	X	X	X		X	X					
Kurstjens S (19)	91		X						X	X			X	X		X	X	X				
Tordjman M (20)	91.8 (89.1–94.6)												X	X								
Gupta-Wright A (21)	83 (82–85)		X		X					X						X				X		
Vieceli T (22)	82.7 (75–90)									X			X				X					
Pardo Lledias J (23)	89 (83.1–94.6)				X			X									X					
Trubiano J (24)	83.6		X		X	X																
Ahmed S (25)	59 (0.45–0.74)									X		X	X			X	X	X				
Elimian KO (26)	Loss of smell in children: 0.56 (0.55– 0.56), either fever or cough in adults: 0.57 (0.56–0.58), and difficulty breathing in the elderly: 0.53 (0.48–0.58).				X																	
Mc Rae AD (27)	Sensitivity: 97.4 (96.4–98.3), Specificity: 95.9 (95.5–96)		X	X	X	X	X	X	X	X	X	X	X	X	X	X	X	X	X	X		

a CK, Creatin Kinase; CRP, C-reactive protein; CT, computerized tomography; CXR, chest X-rays; ECG, electrocardiogram; LDH, lactate dehydrogenase; LYP, blood lymphocyte percentage; LYC, lymphocyte count; RBC, red blood cell; WBC, white blood cell count.

Three of the articles that used AI (12, 15, 18) and four of the studies that developed an algorithm/probability score (19, 20, 26, 27) validated the developed model in an independent sample. In addition, other studies internally validated the model through techniques such as the bootstrap technique (a statistical procedure that resamples a single dataset to create many simulated samples) (21, 22, 24) or a k-fold cross-validation process (an approach that randomly divides the set of observations into k groups, or folds, of approximately equal size; the first fold is treated as a validation set, and the method is fit on the remaining k-1 folds) (13, 14, 17) (Table 1). In accordance with the authors’ conclusions (Table 2), five of the nine studies that did not carry out an external validation remarked the importance of further validation before the application of the model to clinical practice (21–25). Aljame M et al. (16) carried out a validation of a previous score.

### (ii) Studies including patients diagnosed with SARS-CoV-2 infection.

Out of the 23 studies selected for the review, six included patients with a confirmed diagnosis by RT-qPCR in a hospital setting (28–33). Three of the studies used a cross-sectional design (29, 30, 32), one of the studies used a retrospective cohort design (28), one study used an open-cohort design (31), and one was a case-control study (22). The sample size ranged from 114 to 192,778 patients. Four studies used AI (29, 30, 32, 33); Planted TB et al. (33) developed the model in a training set and validated it in an independent set. Two studies internally validated the model through a k-fold cross validation process (30, 31).

Four of the six studies included whole blood tests and biochemical measurements (Tables 2 and 4). Only two of the studies included demographic characteristics. Variables such as imaging test characteristics (CXR/CT) were only included in the Gatti M, et al.’s study (22). AUC levels were higher than those in the studies which included patients with suspected SARS-CoV-2 infection (between 84.2 and 100).

**TABLE 4:** Variables finally included in the model to diagnose infection with SARS-COV-2 in patients diagnosed with COVID-19^*a*^

References	AUC	Demographic (age, sex, race)	Occupation	Contact with COVID-19 patients	Clinical characteristics			Image		Whole blood tests				Biochemical measurements				
					Comorbidities	Symptoms	Days since onset	CT	CXR	RBC	LYP	LYC	WBC	Laboratory routine tests	CRP	LDH	Arterial/venous blood gas	Procalcitonin
Gatti M (28)	87.8						X		X						X	X		
Plante TB (29)	91 (90–92)	X				X								X				
Goreke V (30)	100									X	X	X	X	X	X			
Arpaci I (31)	Accuracy: 84.2									X	X	X	X					X
Marateb HR (32)	87–96	X	X	X	X	X							X	X			X	
Yousif AY (33)	Accuracy: 87									X	X	X	X	X			X	

a CRP, C-reactive protein; CT, computerized tomography; CXR, chest X-rays; LDH, lactate dehydrogenase; LYP, blood lymphocyte percentage; LYC, lymphocyte count; RBC, red blood cell; WBC, white blood cell count.

### (iii) Other studies.

Banerjee A et al. (34) included patients who had samples collected to perform the RT-qPCR technique during a visit to the hospital. However, they did not include data related with patients’ clinical characteristics. They applied machine learning and random forest (a supervised learning algorithm that randomly creates and merges multiple decision trees into one “forest”) to identify SARS-CoV-2 positive patients from full blood counts, and they showed an AUC of 85. Nevertheless, the authors stated that further validation will be required.

### Description of the fulfilment of the STARD criteria.

The average number of criteria fulfilled was 17.6 (maximum, 27 and minimum, 5) over 34 (Table 5 and supplementary material Table 1A and 1B).

**TABLE 5:** Description of the compliance with items included in the checklist of the Standards for Reporting Diagnostic Accuracy Studies guidelines (STARD)

Item from STARD Checklist			Compliance	
			N	(%)
Title or abstract				
	1	Identification as a study of diagnostic accuracy using at least one measure of accuracy (such as sensitivity, specificity, predictive values, or AUC)	12	35.3
Abstract				
	2	Structured summary of study design, methods, results, and conclusions (for specific guidance, see STARD for Abstracts)	16	47.1
Introduction				
	3	Scientific and clinical background, including the intended use and clinical role of the index test	21	61.8
	4	Study objectives and hypotheses	21	61.8
Methods				
Study design	5	Whether data collection was planned before the index test and reference standard were performed (prospective study) or after (retrospective study)	18	52.9
Participants	6	Eligibility criteria	13	38.2
	7	On what basis potentially eligible participants were identified (such as symptoms, results from previous tests, inclusion in registry)	17	50
	8	Where and when potentially eligible participants were identified (setting, location, and dates)	19	55.9
	9	Whether participants formed a consecutive, random or convenience series	12	35.3
Test methods	10a	Index test, in sufficient detail to allow replication	17	50
	10b	Reference standard, in sufficient detail to allow replication	14	41.2
	11	Rationale for choosing the reference standard (if alternatives exist)	10	29.4
	12a	Definition of and rationale for test positivity cut-offs or result categories of the index test, distinguishing prespecified from exploratory	14	41.2
	12b	Definition of and rationale for test positivity cutoffs or result categories of the reference standard, distinguishing prespecified from exploratory	9	26.5
	13a	Whether clinical information and reference standard results were available to the performers/readers of the index test	1	2.9
	13b	Whether clinical information and index test results were available to the assessors of the reference standard	6	17.6
Analysis	14	Methods for estimating or comparing measures of diagnostic accuracy	20	58.8
	15	How indeterminate index test or reference standard results were handled	1	2.9
	16	How missing data on the index test and reference standard were handled	5	14.7
	17	Any analyses of variability in diagnostic accuracy, distinguishing pre-specified from exploratory	13	38.2
	18	Intended sample size and how it was determined	2	5.9
Results				
Participants	19	Flow of participants using a diagram	8	23.5
	20	Baseline demographic and clinical characteristics of participants	18	52.9
	21a	Distribution of severity of disease in those with the target condition	10	29.4
	21b	Distribution of alternative diagnoses in those without the target condition	5	14.7
	22	Time interval and any clinical interventions between index test and reference standard	4	11.8
Test results	23	Cross tabulation of the index test results (or their distribution) by the results of the reference standard	19	55.9
	24	Estimates of diagnostic accuracy and their precision (such as 95% confidence intervals)	20	58.8
	25	Any adverse events from performing the index test or the reference standard	0	0
Discussion				
	26	Study limitations, including sources of potential bias, statistical uncertainty, and generalizability	21	61.8
	27	Implications for practice, including the intended use and clinical role of the index test	19	55.9
Other Information				
	28	Registration no. and name of registry	2	5.9
	29	Where the full study protocol can be accessed	2	5.9
	30	Sources of funding and other support; role of funders	15	44.1

Most of the articles met the two criteria included in the introduction section (61.8%) and detailed the objectives and hypotheses of the study. However, only 35.3% of the articles were identified as a diagnostic accuracy study, and 47.1% included a structured abstract and the scientific and clinical background that justified the study.

In the method section, less than 40% of the studies described the eligibility criteria. For instance, only two of the nine studies that included patients suspected of infection included the days since onset as a variable, and half of the studies included a description of the symptoms. In addition, only two studies included in the review described how the authors estimated the sample size and only one mentioned how indeterminate index test or reference standard results were handled. Of the 23 included studies, less than 20% described if the evaluation of the index test and the reference standard was blind.

In the result section, given the characteristics of the tests evaluated, no study included the description of any adverse effect related with either the index test or the reference standard. Only four (11.8%) studies described time interval between index test and reference standard and five studies (14.7%) included the item related with the distribution of alternative diagnoses in those without the target condition. Only 58.8% of the studies included the precision of the AUC.

Most of the studies did not include a registration number of the study and did not explain if the protocol could be accessed.

## DISCUSSION

The emergence of the pandemic has meant that researchers and journals have made a great effort to generate scientific evidence with available results, often in an accelerated manner. This may have influenced the quality of published studies, increased the risk of bias, and often provided incomplete information. However, poor compliance with the available recommendations for the presentation of results in a scientific article, such as the STARD guidelines (in this review, the mean compliance with the STARD criteria [over 34] was 17.6; range 5–27) may have implications for the care of individuals suspected of having COVID-19 and for the healthcare system (35).

The studies in this review showed high diagnostic accuracy values when more than one test was included, even higher than those included in previous reviews of molecular studies for the detection of COVID-19 (7). A previous review (36) showed how the combination of different diagnostic tests was highly recommended to achieve adequate diagnostic sensitivity and specificity values.

We grouped the studies according to the population, which included symptomatic population with suspected infection, population with a confirmed diagnosis, and others. Diagnostic models should be developed in populations as similar as possible to those in which the test will be applied in practice (37, 38); either in those patients with suspected infection (symptomatic patients or close contact of people with confirmed SARS-CoV-2) or in screening populations. About 70% of the studies included in this review (16 of 23 studies) selected symptomatic patients who presented to a hospital center with suspected infection. Many COVID-19 infections are asymptomatic but can transmit the virus to others. Therefore, early detection of these patients is essential to interrupt the transmission pathway of the virus and control COVID-19. However, given the difficulty of detecting these patients and the fact that routine testing for asymptomatic patients usually includes only one test (usually RT-qPCR), only one of the studies was performed in a population including not only symptomatic patients, but also patients with contact with known COVID-19 positive patient or patients who have traveled abroad, a usual situation in clinical practice (24).

Six of the studies in this review included a population already diagnosed and one study did not specify the clinical characteristics of the patients included. These seven studies (30% of the included studies), therefore, did not provide results that can assist clinicians in identifying the cases they are going to deal with in practice. In a previous review of molecular studies (7), half of the articles included patients with a confirmed diagnosis of COVID-19 infection.

For a diagnostic model to be applied in practice, it must be validated in a sample which is independent from that in which it was developed (39). Only seven of the 16 studies that included symptomatic patients with suspected infection (12, 15, 18–20, 26, 27) and one of the two studies that included already diagnosed patients (33) carried out such external validation. Authors stated the need for further validation of the model in only five of the nine studies that did not include an external validation (21–25). Eight studies (13, 14, 17, 21, 22, 24, 30, 31) carried out internal validation through the application of techniques such as bootstrap or k-fold cross validation. These techniques provide reasonably valid estimates of the diagnostic model, but external validation with substantial sample sizes should not be overlooked (40).

Imaging test results and lymphocyte counts were most frequently included in models diagnosing symptomatic patients with suspected infection. A previous study (41) also showed the diagnostic value of lymphocyte count. To date, imaging has been an essential test in clinical practice for the diagnosis of COVID (42), given that the presence of bilateral pneumonia is an indication of patient risk. However, the studies did not include any information regarding the use of the findings from the imaging tests. Even though chest X-ray and CT chest imaging findings can be useful, they are also understandably nonspecific, can change with disease progression, or have overlap with other viral pneumonias (43). Other variables such as sociodemographic characteristics, presence of comorbidities, or days since symptom onset were only included in less than half of the studies. A previous review assessing symptoms associated with infection did not identify any useful combination of symptoms that could be reliably associated with the presence of infection (11). One of the studies (12) included the first RT-qPCR performed in the model, while establishing the final result of the RT-qPCR as the gold standard. A previous review (44) indicated the frequency of false-negative results of the first RT-qPCR; their inclusion in the model may lead to a high risk of diagnostic verification bias if only some of the participants who received this first RT-qPCR with a particular result received the standard reference test (45).

Ten of the 23 included studies applied artificial intelligence methods, nine studies focused on symptomatic patients (19–27), four studies on patients already diagnosed (29, 30, 32, 33) and in one study, the clinical characteristics of the included patients are unspecified (34). Although an effort is being made to develop guidelines for the description of AI-based diagnostic studies (46), given the novelty of the application of this procedure, they have not yet been fully developed. This may also have contributed to the lack of compliance of these studies with the STARD criteria.

The mean compliance with the STARD criteria was 17.6 over 34 (range 5–27). Given the characteristics of the studies, both the diagnostic model and the gold standard were performed in a short span of time, which limited errors arising from changes in the patients' disease status. However, this also led to lower compliance with the criteria related with the independent evaluation of the two tests. Within the results section, a large majority of studies did not describe the flow diagram of patients, or the distribution of disease severity and other alternative diagnoses in those patients with and without the disease under study. This can limit the usefulness of the study for clinicians who are trying to apply the results in practice. Only 35% of the evaluated studies included a description of the patient selection process (consecutive, randomized or other) and more than 60% of them did not describe the patient inclusion criteria. Although this bias has also been described in the evaluation of molecular tests for COVID-19 (47), in this review this description represents an improvement on previous reviews, where 98% of the studies were at high risk of selection bias (48). The articles did not show sample size determination, nor did they describe how to deal with indeterminate or missing results. Finally, almost all the studies failed to show the prior registration of the study, as well as whether the protocol was accessible. This is an important question, as it would allow us to establish whether the strategy tested was proposed prior to seeing the results. If researchers only report on the combinations of tests that obtain high values of diagnostic accuracy, they run the risk of developing models which fit the data (overfitting) but have limited clinical value. This is especially relevant when coupled with the absence of validation in an independent sample.

The limitations of this study are mainly related to the literature search. Although the search was conducted in different databases (PubMed, Embase, and the bioRxiv and medRxiv databases), given the wide dissemination of research during the pandemic, it is possible that we have missed some relevant studies. Given the urgency to develop new diagnostic strategies in the COVID-19 pandemic, there may have been a bias related to the publication of only those results that show a significant finding.

### Conclusions.

According to this systematic review, the inclusion of more than one diagnostic test in the diagnostic process for COVID-19 infection shows high diagnostic accuracy values. Imaging characteristics, patients’ symptoms, demographic characteristics, and lymphocyte count were the variables most frequently included in the diagnostic models. However, there are some aspects to be improved; developed models should be externally validated before reaching conclusions on their utility in practice. In addition, it is important to bear in mind that the test should be evaluated in populations as similar as possible to those in which it will be applied in practice. Applying existing recommendations for the development of diagnostic tests, including following STARD or similar guidelines in the case of AI studies, will improve the reporting of diagnostic evaluation studies.

## MATERIALS AND METHODS

This systematic review was conducted according to the recommendations of the Preferred Reporting Items for Systematic Reviews and MetaAnalyses (PRISMA) (49).

### Eligibility criteria.

Original research articles (published from 1 January 2020 to 28 February 2022) were selected. We included studies whose primary objective was to evaluate a diagnostic strategy to detect the presence of SARS-CoV-2 infection before getting a molecular diagnosis; the diagnostic strategy was designed for use in clinical practice or a public health context, and accuracy rates had to be provided either in terms of sensitivity and specificity, the likelihood ratio, or ROC curve. Our search was limited to articles written in English or Spanish. We excluded articles that focused on the evaluation or validation of a single individual diagnostic test or the comparison between different individual diagnostic tests.

### Search strategy.

We conducted a comprehensive and sensitive search based on search terms developed for the COVID-19 Open Access Project by researchers and librarians at the Institute of Social and Preventive Medicine of the University of Bern (https://www.ispm.unibe.ch/about_us/news/new_searchable_living_evidence_in_covid_19_open_access_project/index_eng.html). The search for articles included PubMed, Embase and preprints indexed in the bioRxiv and medRxiv databases.

The following search terms were applied to uncover studies of diagnostic accuracy: “Sensitivity and Specificity ”[Mesh], “Sensitivity” or “Specificity,” “Area Under Curve” [Mesh],or “Area under curve,” “ROC Curve” [Mesh], or “ROC,” “Diagnostic Accuracy,” “Likelihood Ratio” and “Severe Acute Respiratory Syndrome Coronavirus 2” [Supplementary Concept], “COVID- 19” [Supplementary Concept], “Coronavirus” or “Corona Virus,” “HCoV” or “nCoV,” “2019 CoV,” “Covid,” “Covid19,” “Severe Acute Respiratory Syndrome Coronavirus 2,” “SARS-CoV2,” “SARS-CoV 2,” or “SARS Coronavirus 2”.

### Study selection.

We included original articles on a diagnostic strategy to detect the presence of SARS-CoV-2 infection, which included the combination of at least two diagnostic tests. A screening was performed to exclude editorials, letters to the editor, and any study different from an original research report.

Two reviewers (P.C.-M and B.L.) independently screened each reference title and abstract (if available) for relevance to the review. Disagreements were resolved by discussion and consensus with a third reviewer (E.Ch.R. and L.A.P.). We then, reviewed the complete article of those selected in this first round. In the second round, two reviewers independently applied the selection criteria. Studies in which the authors did not specifically perform an assessment of the diagnostic accuracy of a diagnostic process involving the combination of more than one test were excluded.

For the inclusion of the studies, it was established that the concordance assessment between these authors (κ-index) should be greater than 0.60.

### Data extraction.

The following variables were extracted from each of the included articles: year of publication, country of origin, study design, clinical setting, study objective (diagnosis or screening), characteristics of the study population (sex, age, symptoms, and sample size), tests included in the diagnostic strategy, results of the diagnostic tests and authors' conclusions. Data extraction was performed independently by two researchers (P.C.-M. and B.L.) and then discrepancies were discussed independently with a third author (E.Ch.R. or L.A.P.).

Two reviewers (P.C.-M. and B.L.) also independently applied the STARD criteria and discrepancies were discussed with a third author (E.Ch.R. or L.A.P.). The results of this assessment are summarized in the supplementary material.

Concordance was also analyzed using the kappa index of both the extraction of study variables and the application of the STARD guideline.
