# Supplementary material for: Evaluation of Diagnostic Strategies for Identifying SARS-CoV-2 Infection in Clinical Practice: a Systematic Review and Compliance with the Standards for Reporting Diagnostic Accuracy Studies Guideline (STARD)
Source: Microbiol Spectr. 2022 Jun 14;10(4):e00300-22. doi: 10.1128/spectrum.00300-22 (PMC9430610; doi:10.1128/spectrum.00300-22)
Supplement: Supplemental file 1 — Supplemental material. Download spectrum.00300-22-s0001.pdf, PDF file, 0.2 MB [file spectrum.00300-22-s0001.pdf]

Supplementary material table 1A. Compliance with the items included in the STARD checklist detailed for the studies with symptomatic patients with suspected COVID-19 infection.

	Guo X (13)	Langer T (14)	Hermans JJR (15)	Yang HS (16)	Aljame M (17)	Tschoellitsch T (18)	Du R (19)	Kurstjens S (20)	Tordjman M (21)	Gupta-Wright A (22)	Vieceli T (23)	Pardo Lledias J (24)	Trubiano JA (25)	Ahmed S (26)	Elimian KO (27)	McRae AD (28)
**Title or abstract**																
1	Y	Y	Y	Y	N	N	N	Y	Y	Y	Y	Y	Y	N	N	N
**Abstract**																
2	Y	Y	Y	Y	N	Y	N	Y	Y	Y	Y	Y	Y	Y	Y	Y
**Introduction**																
3	Y	Y	Y	Y	Y	N	Y	Y	Y	Y	Y	Y	N	Y	Y	Y
4	Y	Y	Y	Y	Y	Y	Y	N	Y	Y	Y	Y	N	Y	Y	Y
**Methods**																
Study design																
5	N	Y	Y	N	Y	N	Y	Y	Y	Y	Y	Y	Y	Y	Y	Y
Participants																
6	N	Y	Y	N	N	N	Y	Y	Y	Y	Y	Y	N	N	Y	Y
7	Y	Y	Y	Y	Y	N	Y	Y	Y	Y	Y	Y	Y	N	Y	Y
8	Y	Y	N	Y	Y	Y	Y	Y	Y	Y	Y	Y	Y	N	Y	Y
9	N	Y	Y	N	Y	Y	Y	N	N	Y	Y	N	N	N	Y	Y
Test methods																
10a	Y	N	Y	Y	Y	N	N	Y	Y	Y	Y	Y	Y	Y	Y	Y
10b	N	Y	Y	Y	Y	N	N	N	Y	Y	Y	Y	N	N	Y	Y
11	N	N	Y	N	Y	N	Y	Y	Y	Y	Y	N	N	N	N	N
12a	Y	N	N	N	Y	N	Y	Y	Y	Y	Y	N	N	Y	Y	Y

12b	N	Y	N	N	Y	N	Y	Y	Y	Y	N	N	N	N	Y	N
13a	N	N	N	N	N	N	N	N	N	Y	N	N	N	N	N	N
13b	N	N	N	N	N	N	N	N	N	Y	N	N	N	Y	Y	N
Analysis																
14	Y	Y	Y	Y	Y	N	Y	Y	Y	Y	Y	Y	Y	N	Y	Y
15	N	N	N	N	N	N	Y	N	N	N	N	N	N	N	N	N
16	N	N	N	Y	N	N	Y	N	N	N	N	N	N	N	Y	N
17	Y	Y	N	Y	N	N	Y	Y	Y	N	Y	Y	Y	N	Y	N
18	N	N	N	N	N	N	N	N	N	N	N	N	N	N	Y	Y
**Results**																
Participants																
19	N	N	Y	Y	N	N	N	N	Y	Y	N	N	N	N	Y	Y
20	Y	Y	Y	Y	N	Y	Y	Y	Y	Y	Y	Y	Y	N	Y	Y
21a	N	Y	Y	Y	N	N	N	Y	Y	Y	N	Y	Y	N	Y	N
21b	Y	N	N	N	N	N	Y	N	N	Y	N	Y	N	N	N	N
22	N	N	Y	N	N	N	Y	N	N	Y	N	N	N	NA	NA	N
Test results																
23	Y	Y	Y	Y	Y	N	Y	Y	Y	Y	Y	Y	Y	Y	N	Y
24	Y	N	Y	Y	Y	Y	Y	N	Y	Y	Y	Y	Y	Y	Y	Y
25	N	N	N	N	N	N	N	N	N	NA	N	N	N	N	N	N
**Discussion**																
26	Y	Y	Y	Y	Y	Y	Y	Y	Y	Y	Y	Y	Y	Y	Y	Y
27	Y	Y	Y	Y	Y	N	Y	Y	N	Y	Y	Y	Y	Y	Y	Y
**Other information**																
28	N	N	N	N	N	N	N	N	N	N	N	Y	N	Y	N	N

29	Y	N	N	N	N	N	N	N	N	N	N	N	N	Y	N	N
30	Y	Y	N	Y	N	N	N	Y	Y	Y	N	Y	Y	Y	Y	Y
**Total Yes**	**18**	**19**	**20**	**19**	**16**	**7**	**21**	**19**	**22**	**27**	**20**	**21**	**15**	**14**	**24**	**20**

Supplementary material table 1B. Compliance with the items included in the STARD checklist detailed for the studies with patients with confirmed COVID-19 infection and other.

	Gatti M (29)	Goreke V (30)	Arpaci I (31)	Marateb HR (32)	Yousif AY (33)	Plante TB (34)	Banerjee A (35)
**Title or abstract**							
1	Y	N	N	N	N	Y	N
**Abstract**							
2	Y	N	N	N	N	Y	N
**Introduction**							
3	Y	Y	Y	Y	Y	Y	Y
4	Y	Y	Y	Y	Y	Y	Y
**Methods**							
Study design							
5	Y	N	N	Y	Y	Y	Y
Participants							
6	Y	N	N	Y	N	Y	N
7	Y	N	N	Y	N	N	Y
8	Y	N	Y	Y	N	Y	Y
9	Y	N	N	N	N	Y	Y
Test methods							
10a	Y	N	N	Y	N	Y	Y
10b	Y	N	N	Y	N	Y	Y
11	Y	N	N	Y	N	N	Y
12a	Y	N	N	Y	Y	N	Y

12b	N	N	N	Y	N	N	Y
13a	N	N	N	N	N	N	N
13b	N	N	Y	Y	Y	N	N
Analysis							
14	Y	Y	Y	Y	Y	Y	N
15	N	N	N	N	N	N	N
16	N	N	Y	Y	N	N	N
17	N	N	N	Y	N	Y	Y
18	N	N	N	N	N	N	N
**Results**							
Participants							
19	Y	N	N	N	N	Y	N
20	Y	N	Y	Y	N	Y	N
21a	N	N	N	Y	N	N	N
21b	N	N	N	N	N	N	Y
22	Y	N	NA	N	N	N	N
Test results							
23	N	Y	N	Y	Y	Y	Y
24	N	Y	Y	Y	Y	Y	Y
25	N	N	N	N	N	N	N
**Discussion**							
26	Y	N	Y	Y	Y	Y	N
27	N	N	Y	Y	Y	Y	Y
**Other information**							
28	N	N	N	N	N	N	N

29	N	N	N	N	N	N	N
30	Y	N	N	Y	N	Y	Y
**Total Yes**	**19**	**5**	**10**	**22**	**10**	**19**	**17**
